# Supplementary material for: Targeting the capsule of Klebsiella pneumoniae with a cationic CR3-binding protein enhances phagocytosis and promotes bacterial clearance and survival in a mouse sepsis model
Source: Front Immunol. 2026 Oct 2;17:1934439. doi: 10.3389/fimmu.2026.1934439 (PMC13671991; doi:10.3389/fimmu.2026.1934439)
Supplement: Supplementary file 1 [file DataSheet1.pdf]

# SUPPLEMENTARY MATERIAL

## TARGETING THE CAPSULE OF *KLEBSIELLA PNEUMONIAE* WITH A CATIONIC CR3-BINDING PROTEIN ENHANCES PHAGOCYTOSIS AND PROMOTES BACTERIAL CLEARANCE AND SURVIVAL IN A MOUSE SEPSIS MODEL

Nataly P. Podolnikova, Iryna Klymenko, James Alagna, Cyrus Diercksmeier, Arnat Balabiyev, David Richardson, and Tatiana P. Ugarova

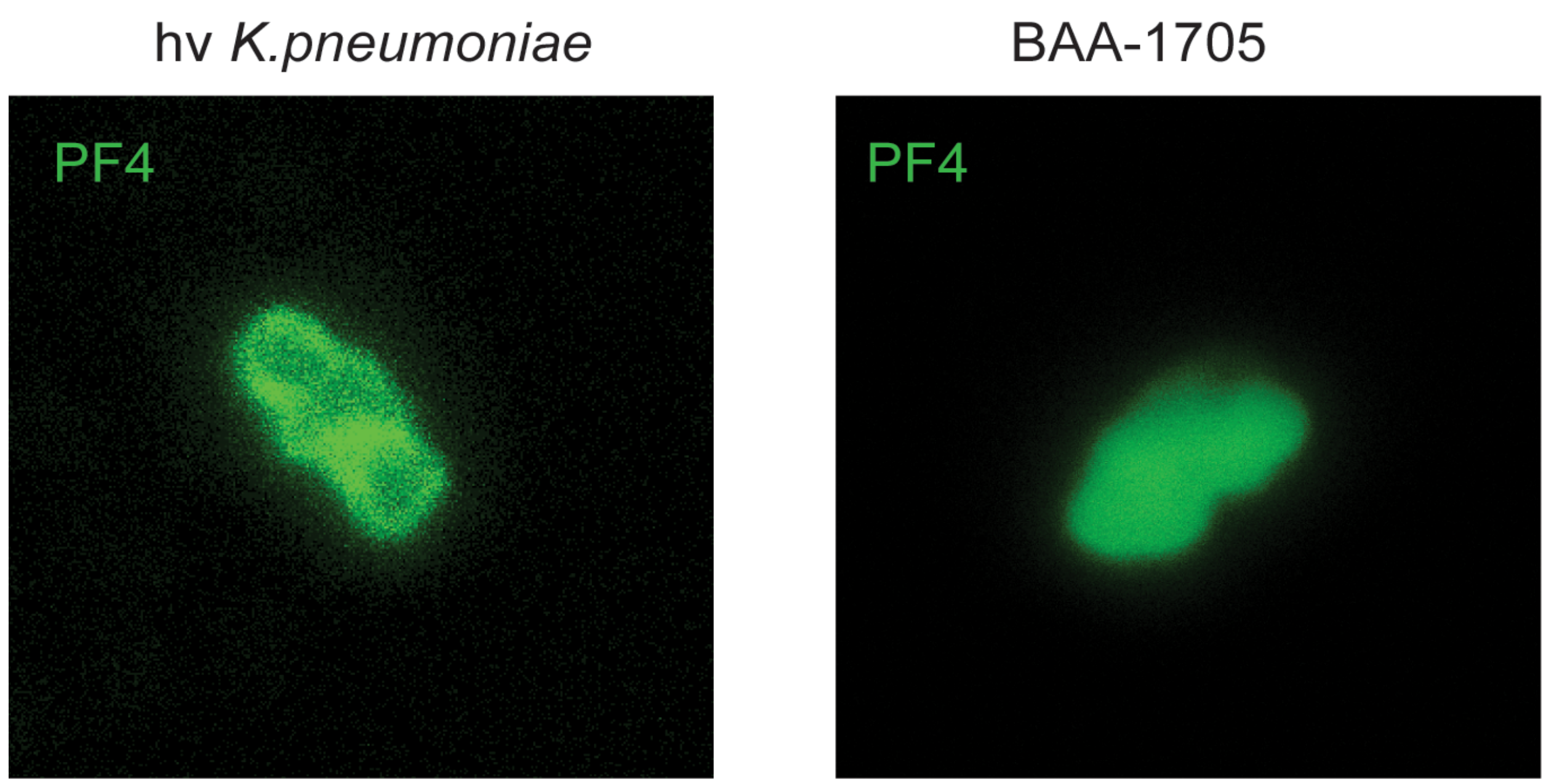


Fig. S1

**Figure S1.** Binding of rdPF4 to hv (K2 strain) and antibiotic-resistant *K. pneumoniae* strains. **A**, **B,** Confocal images showing rPF4 binding to the surface of K2 type of *K. pneumoniae* (A) and BAA-1705 *K. pneumoniae* (B). rPF4 (100 μg/ml) was incubated with bacteria for 60 minutes at 37 ºC. After removing unbound rPF4, bacteria were incubated with rabbit polyclonal anti-PF4 antibody (1:250) for 30 minutes at 22 ºC, followed by incubation with goat anti-rabbit Alexa Fluor 488-conjugated antibody.

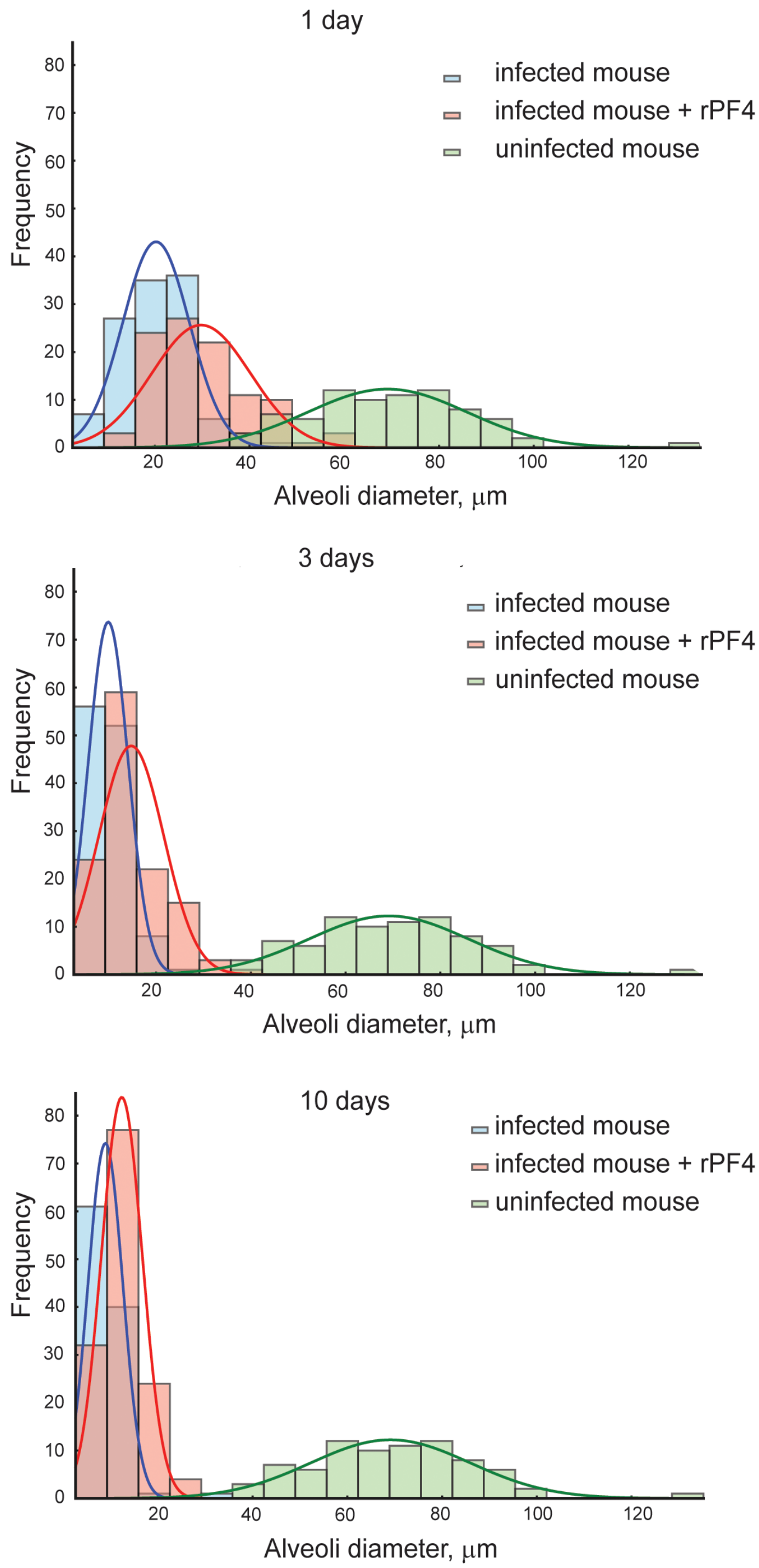


Fig. S2

**Figure S2.** Frequency distribution of the total alveolar diameter in lungs of uninfected, infected, and infected mice treated with rPF4 on days 1, 3, and 10 after infection with low-virulence *K. pneumoniae.*

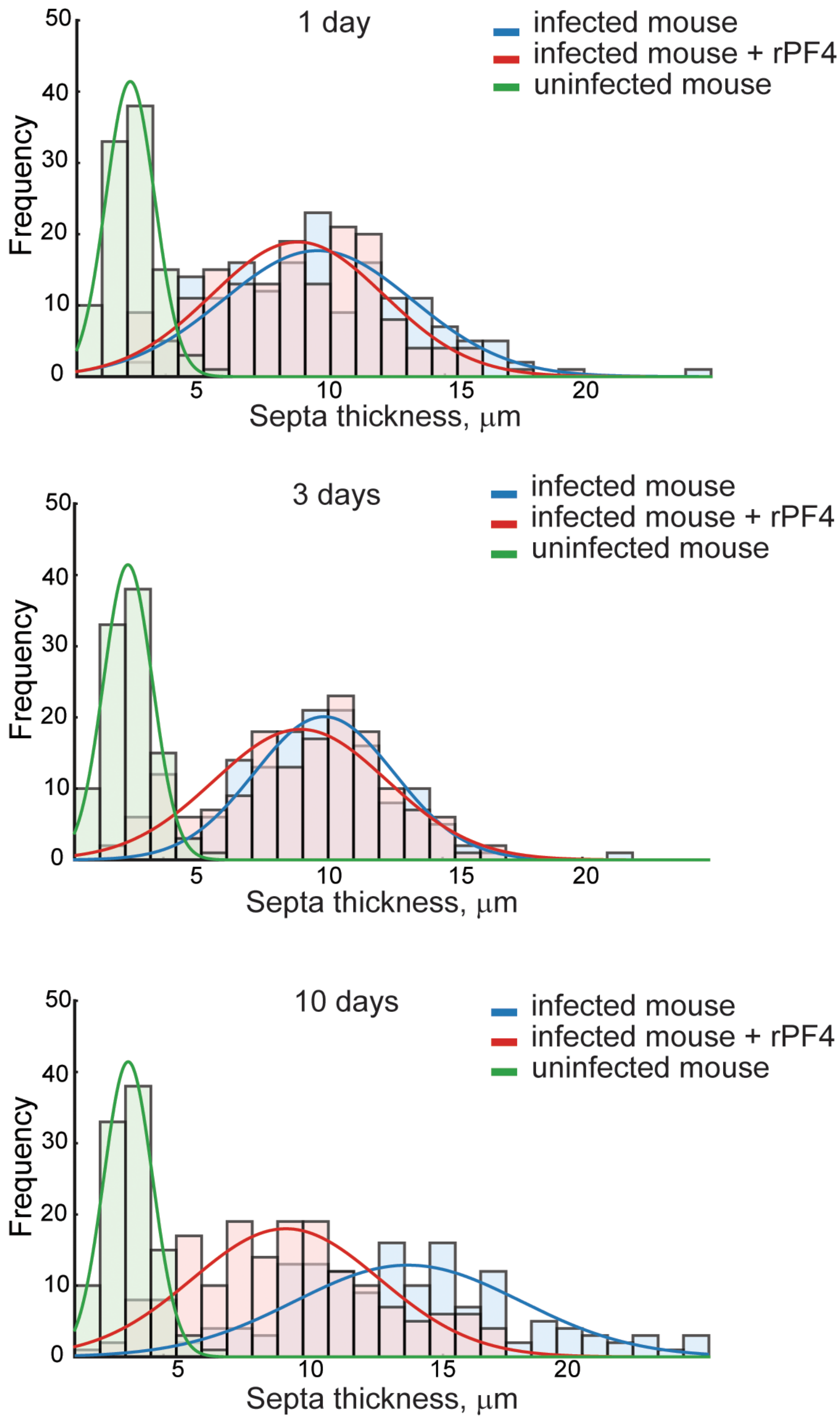


Fig. 3S

**Figure S3.** Frequency distribution of septal thickness in the lungs of uninfected, infected, and rPF4-treated mice 1, 3, and 10 days after infection with low-virulence *K. pneumoniae*.

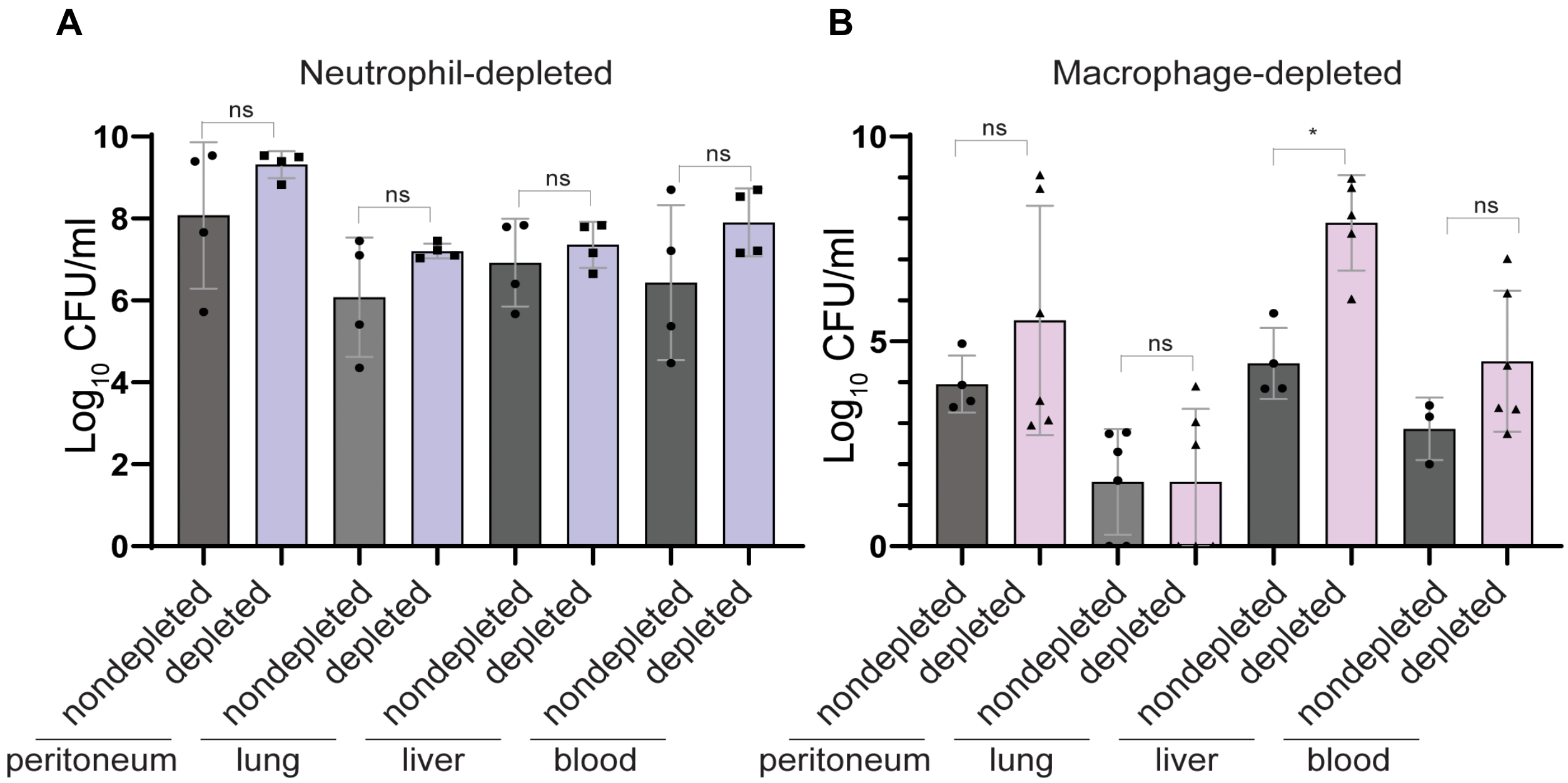


Fig. S4

**Figure S4.** Counts of bacteria in the peritoneum, lungs, liver, and blood 24 hours after infection with $3x10^7$ CFU of lv*Kp* in neutrophil-depleted mice (n= 4) and with $1x10^7$ CFU in macrophage-depleted mice (n=6). Data are presented as means ± S.D. of log-transformed CFU/ml values. Statistical significance was assessed using a two-tailed paired t-test on the log-transformed data.

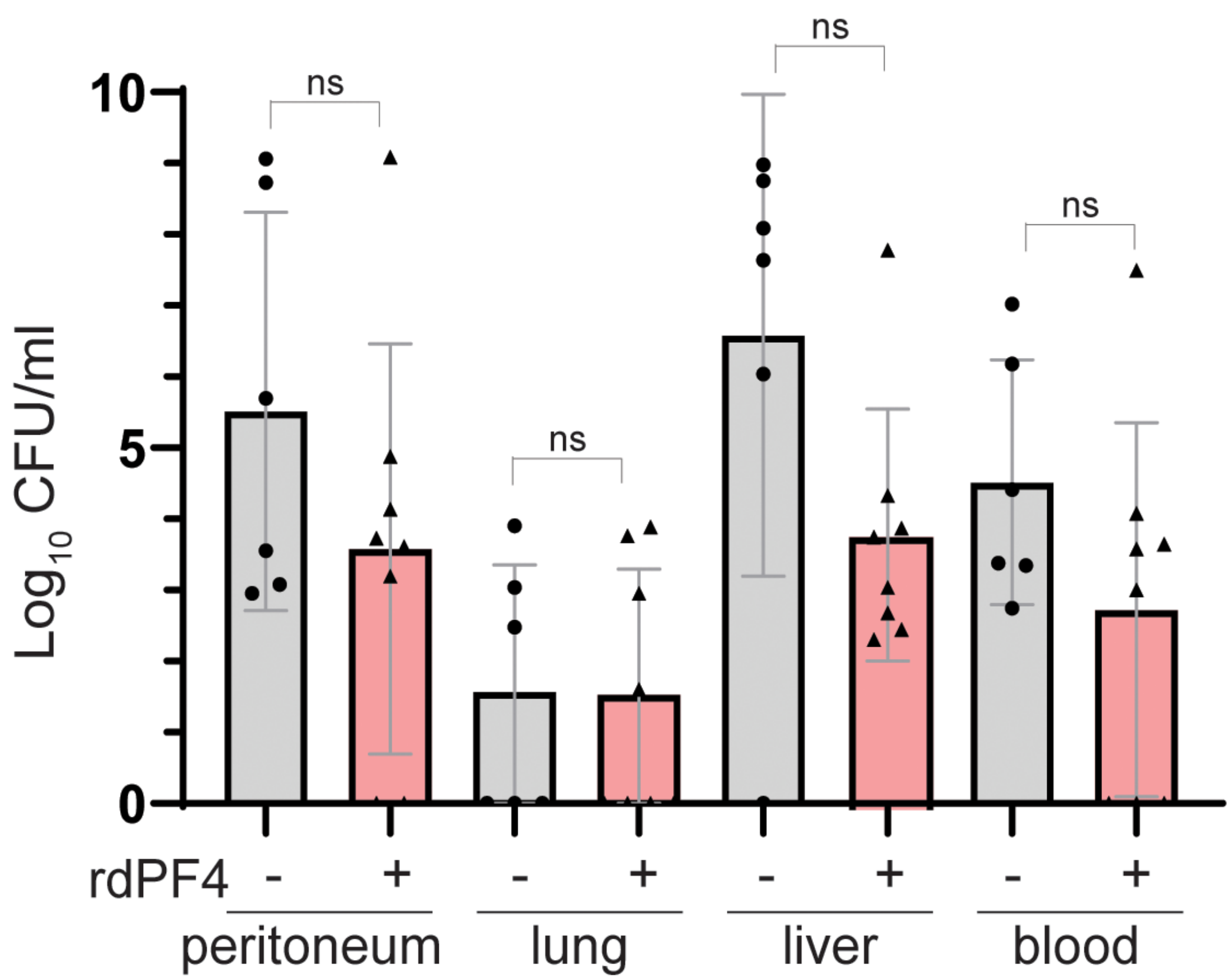


Fig S5

**Figure S5.** Effect of rPF4 on clearance of lv*Kp* in macrophage-depleted mice. Mice (n=14) received a retro-orbital injection of 0.2 mL of Clodronate-containing liposomes, followed 24 hours later by an intraperitoneal injection of another 0.2 mL of Clodronate-containing liposomes. Lv*Kp* ($1x10^7$) was administered on day 3 after the initial liposome treatment. Following infection, 8 mice received rPF4 (12 µg/mouse). The effect of rPF4 on bacterial clearance in the peritoneum, blood, lungs, and liver in macrophage-depleted mice was assessed 24 hours after infection. Peritoneal lavage and blood samples were serially diluted and plated on LB agar, then incubated for 16 hours at 37 °C. Lung and liver tissues were weighed, homogenized, and passed through a 100-µm cell strainer. The collected samples were serially diluted, plated on LB agar, and incubated for 16 hours at 37 °C; colonies were then counted. Data are presented as means ± S.D. of log-transformed CFU/ml values from 6 to 8 mice per group.

| | | | | | | | | | | | |
|---|---|---|---|---|---|---|---|---|---|---|---|
| rPF4 | 200ug/ml | 100 | 50 | 25 | 12.5 | 6.25 | 3.13 | 1.6 | 0.78 | 0.04 | 0.02 |
| Ampiciline | 1024ug/ml | 512 | 256 | 128 | 64 | 32 | 16 | 8 | 4 | 2 | 1 |
| Kanamycin | 256ug/ml | 128 | 64 | 32 | 16 | 8 | 4 | 2 | 1 | 0.5 | 0.25 |
| Ceftriaxone | 256ug/ml | 128 | 64 | 32 | 16 | 8 | 4 | 2 | 1 | 0.5 | 0.25 |
| Penyciline/streptomiocyne | 256ug/ml | 128 | 64 | 32 | 16 | 8 | 4 | 2 | 1 | 0.5 | 0.25 |
| Meropenem | 256ug/ml | 128 | 64 | 32 | 16 | 8 | 4 | 2 | 1 | 0.5 | 0.25 |
| Tetracycline | 256ug/ml | 128 | 64 | 32 | 16 | 8 | 4 | 2 | 1 | 0.5 | 0.25 |
| Gentamicine | 256ug/ml | 128 | 64 | 32 | 16 | 8 | 4 | 2 | 1 | 0.5 | 0.25 |

Fig S6

**Figure S6**. Minimum Inhibitory Concentration (MIC) of 7 antibiotics and rPF4 against the BAA-1705 strain.
